# Prevalence and genetic diversity of *Aeromonas veronii* isolated from aquaculture systems in the Poyang Lake area, China

**DOI:** 10.3389/fmicb.2022.1042007

**Published:** 2022-12-12

**Authors:** Xiandong Xu, Huiyun Fu, Guoyuan Wan, Jiangfeng Huang, Zhiyong Zhou, Yi Rao, Lihui Liu, Chungen Wen

**Affiliations:** ^1^College of Life Science, Education Ministry Key Laboratory of Poyang Lake Environment and Resource Utilization, Nanchang University, Nanchang, China; ^2^Fisheries Research Institute of Jiangxi Province, Nanchang, China; ^3^Key Laboratory of Fishery Drug Development, Ministry of Agriculture and Rural Affairs, Key Laboratory of Aquatic Animal Immune Technology, Pearl River Fisheries Research Institute, Chinese Academy of Fishery Sciences, Guangzhou, China

**Keywords:** *Aeromonas veronii*, genetic diversity, virulence genes, antimicrobial resistance genes, Poyang Lake area

## Abstract

The area around Poyang Lake is the main aquaculture area in Jiangxi Province, China, and an important base for the supply of freshwater aquatic products. Aquaculture in the Poyang Lake area is severely threatened by diseases caused by bacterial pathogens, and *Aeromonas veronii* has been the main pathogen in recent years. In this paper, ERIC-PCR genotyping, virulence gene and antimicrobial resistance gene detection, and drug susceptibility tests were carried out on 46 *A. veronii* isolates obtained from aquaculture systems in the Poyang Lake area from 2016 to 2020. The results showed that the *A. veronii* strains in the Poyang Lake area had high genetic diversity, and 46 strains produced 36 ERIC genotypes. There were no geographical and temporal differences in the cluster analysis results and no dominant clones. All 13 virulence genes tested were detected, and all isolates had harbored 2 or more virulence genes, with a maximum of 12 virulence genes detected. Among the 22 antimicrobial resistance genes selected, 15 were detected; 97.8% of the isolates contained 2 or more antimicrobial resistance genes, with a maximum of 9 antimicrobial resistance genes. Drug susceptibility tests showed that some strains were resistant to a variety of traditionally effective drugs for *Aeromomas,* such as enrofloxacin and florfenicol. This study provides a reference for exploring the impact of aquaculture in the Poyang Lake area on public health.

## Highlights

- *Aeromonas veronii* was the main pathogen of aquaculture in the Poyang Lake area, and had high genetic diversity.
- There was great variation in virulence genes and antimicrobial resistance gene occurrence, possession and distribution among *Aeromonas veronii* isolates.
- The distribution frequency of virulence genes in clinical strains was higher than that in environmental strains.
- The distribution frequency of antimicrobial resistance genes showed the opposite trend to the virulence genes.
- Floods or the circulation of fry may accelerate *Aeromonas veronii* gene exchange in Poyang Lake area.

## Introduction

Poyang Lake, located in northern Jiangxi Province, is the largest freshwater lake in China and the largest wetland reserve in Asia. Due to its rich aquaculture resources, it has become an important aquaculture area in Jiangxi Province and even in China (Wang et al., 2020). The aquaculture in this area mainly produces grass carp (*Ctenopharyngodon idella*), silver carp (*Hypophthalmichthys molitrix*), bighead carp (*Aristichthys nobilis*) and Pengze crucian carp (*Carassius auratus* var. Pengze). The main cultivation method applied in the Poyang Lake area is intensive pond culture. However, the growth of the aquaculture industry in this region has resulted in some health problems. Common pathogens include bacteria, viruses and parasites. Among the various pathogens, bacteria are the main pathogens and are closely related to human health. Some aquatic bacteria can cause zoonoses that directly endanger human health, and more importantly, some can harm human health through the horizontal transfer of virulence genes and antimicrobial resistance (AMR) genes (Carnelli et al., 2017; Rahman et al., 2020).

The major bacterial infections of freshwater fish generally involve one of several species of the genus *Aeromonas*, such as *A. veronii* and *A. hydrophila* (Fernández-Bravo and Figureueras, 2020). *A. veronii* is the primary pathogen isolated from freshwater fish in South China, including Jiangxi Province (Ran et al., 2018; Xu et al., 2018, 2019, 2021; Gao et al., 2020; Rao et al., 2020; Wang et al., 2021). The cultured fishes in the Poyang Lake area are also affected by *A. veronii*, which is the most frequently detected bacterium in freshwater (Ran et al., 2018; Pessoa et al., 2020). *A. veronii* is widely distributed and has been isolated from diseased aquatic animals and aquatic systems in multiple countries. It has a variety of hosts and can live in humans, aquatic animals, human food and the environment, posing risk to humans (Fernández-Bravo and Figureueras, 2020). Thus, it is necessary to carry out genetic and epidemiological studies of *A. veronii* to prevent diseases caused by this pathogen.

*A. veronii* is recognized as an opportunistic pathogen (Fernández-Bravo and Figureueras, 2020). Identifying the epidemiological relationships of *A. veronii* strains is important for understanding the behaviour of this microorganism in human infections (Martínez-Murcia et al., 2000). A number of typing methods are applied to *Aeromonas* strains for epidemiological purposes (Łaganowska and Kaznowski, 2004; Naviner et al., 2011; Fernández-Bravo and Figureueras, 2020). Compared with other typing methods, enterobacterial repetitive intergenic consensus–polymerase chain reaction (ERIC-PCR), a DNA-based typing method is more rapid and economical, is technically simpler and has a higher discriminatory power for determining the distribution of a single strain among individuals (Xu et al., 2017).

The virulence of *Aeromonas* results in potential damage (Gonçalves Pessoa et al., 2019), and it is impossible to establish a hierarchical classification of virulence factors according to their role in the disease process (Aguilera-Arreola et al., 2007). Therefore, it is necessary to continuously survey the presence of several accepted virulence factors in *A. veronii* isolates, this monitoring is also important for understanding both *A. veronii* pathogenesis and the epidemiology of *A. veronii* infections (Aguilera-Arreola et al., 2007).

Antimicrobials are occasionally used in feed or directly in water in aquaculture systems for disease prevention and treatment in China (Liu et al., 2017). Antibiotics boost the risk of emerging AMR in aquatic bacteria, which is commonly caused by AMR genes. Antimicrobial-resistant bacteria and AMR genes result in biological contamination of the environment (Carnelli et al., 2017). In aquatic environments, antibiotic resistance genes may spread horizontally and be transferred to bacteria colonizing humans and other animals through food and drinking water (San, 2018; Sabbagh et al., 2021).

To our knowledge, there have been no reports characterizing the genetic diversity, virulence and AMR of *A. veronii* in the Poyang Lake area. In this study, the genetic diversity and virulence and AMR genes prevalence in *A. veronii* strains isolated from aquaculture systems in the Poyang Lake area were examined. The results of this study are of great significance for improving the food safety assessment of freshwater aquatic products and understanding its relevance to animal and public health.

## Materials and methods

### Bacterial strains and growth conditions

**Figure 1:**
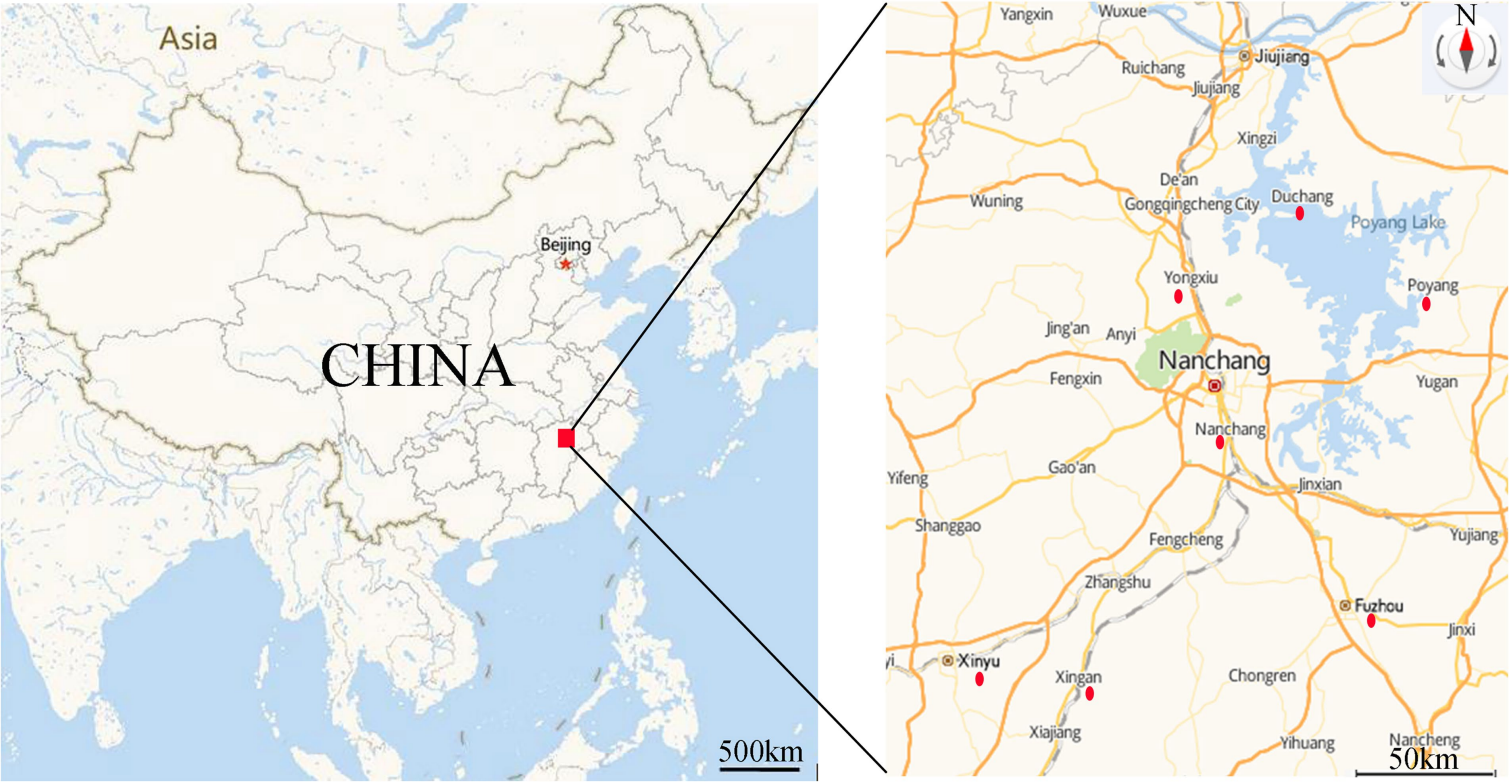
Sampling area and location of sampling sites. Red dot indicated the sampling sites. (https://lbs.amap.com/demo/javascript-api/example/map/map-english/).

A total of 46 strains were previously isolated from diseased fish, aquaculture pond water and sediment from the Poyang Lake area, Jiangxi Province, China, in 2016–2020. All strains were identified as *A. veronii* (Xu et al., 2018, 2019, 2021; Rao et al., 2020) and were stored in broth medium containing 25% glycerol (v/v) at −80°C. The sampling sites were illustrated in Figure 1, and the sources and origins of the *A. veronii* isolates were listed in Table 1.

**Table 1:** Characteristics of 46 *A. veronii* isolates from mariculture systems.

Name of isolate	Source	City	Year of isolation	Genbank no.
BL16103	*Ctenopharyngodon idellus*	Nanchang	2016	KY767513
BL16104	*C. idellus*	Nanchang	2016	KY767536
BL16105	*C. idellus*	Nanchang	2016	KY767517
NX16104	*C. idellus*	Nanchang	2016	KY767507
JX16102	*C. idellus*	Nanchang	2016	KY767538
CCG1	*C. idellus*	Nanchang	2016	KY767539
CCG2	*C. idellus*	Nanchang	2016	KY767540
PYLGC	*C. idellus*	Poyang	2016	KY767542
PYG3	*C. idellus*	Poyang	2016	KY767545
PYG1	*C. idellus*	Poyang	2016	KY767544
DC1S-1	Aquacultural water	Duchang	2017	MF716677
DC2-2-1	Aquacultural water	Duchang	2017	MF716700
DC2S-2	Aquacultural water	Duchang	2017	MF716667
JX1-2-1	Aquacultural water	Nanchang	2017	MF716698
JX1-2-2	Aquacultural water	Nanchang	2017	MF716707
JX3-2-2	Aquacultural water	Nanchang	2017	MF716703
JX3N-1	Pond sediment	Nanchang	2017	MF716690
XG1-2-1	Aquacultural water	Xingan	2017	MF716705
XG2S-1	Aquacultural water	Xingan	2017	MF716693
XG2S-2	Aquacultural water	Xingan	2017	MF716665
XG3-1-1	Aquacultural water	Xingan	2017	MF716697
FZ1SG	*Paramisgurnus dabryanus*	Fuzhou	2017	MF716715
FZ1SY	Aquacultural water	Fuzhou	2017	MF716714
FZ2N	*P. dabryanus*	Fuzhou	2017	MF716720
FZ3SY	aquacultural water	Fuzhou	2017	MF716718
FZQP	*P. dabryanus*	Fuzhou	2017	MF716717
FZQPS	aquacultural water	Fuzhou	2017	MF716712
FZSY	*P. dabryanus*	Fuzhou	2017	MF716721
LJLC	*Hypophthalmichthys molitrix*	Nanchang	2019	MW116752
LTJC	*Carassius auratus var. pengze*	Nanchang	2019	MW116753
LTFS7	*C. idellus*	Nanchang	2019	MW116754
LTFS5	*C. idellus*	Nanchang	2019	MW116757
LTFS2	*C. idellus*	Nanchang	2019	MW116758
LTCC	*C. idellus*	Nanchang	2019	MW116759
LTFS6	*C. idellus*	Nanchang	2019	MW116760
LTFS1	*C. idellus*	Nanchang	2019	MW116761
LTFS4B	*C. idellus*	Nanchang	2019	MW116762
LTFS3	*C. idellus*	Nanchang	2019	MW116763
LTFS4	*C. idellus*	Nanchang	2019	MW116764
YXJ	*C. auratus var. pengze*	Yongxiu	2019	MW116765
YXCY	*C. idellus*	Yongxiu	2019	MW116766
YXC	*C. idellus*	Yongxiu	2019	MW116767
XYY	*Aristichthys nobilis*	Xinyu	2018	MZ414185
XYB	*Culter alburnus*	Ximyu	2018	MZ414186
HML2	*H. molitrix*	Nanchang	2020	MZ414187
HMH2	*Aristichthys nobilis*	Nanchang	2020	MZ414188

### ERIC-PCR

The isolated strains of *A. veronii* were grown overnight at 28°C on nutrient agar plates (Beijing Land Bridge Technology Co., Ltd., China). Chromosomal DNA of the *A. veronii* strain was extracted by a TaKaRa Mini BEST Bacteria Genomic DNA Extraction Kit, version 3.0 (TaKaRa, Japan). ERIC-PCR was performed in a reaction system that had a final volume of 25 μl and contained 2.5 μl Ex Taq buffer (10×), 2.5 mM MgCl_2_, 200 mM each deoxynucleoside triphosphate (dNTPs), 10 pmol each of the ERIC1 primer (5ʹ-ATGTAAGCTCCTGGGGATT CAC-3ʹ) and ERIC2 primer (5ʹ-AAGTAAGTGACTGGGGTGA GCG-3ʹ), 50 ng extracted DNA and 1 U Ex Taq DNA polymerase (TaKaRa, Japan). Amplification was performed in an Eppendorf T-Gradient thermoblock (Eppendorf, Germany). The PCR conditions and electrophoresis observation method followed those of Xu et al. (2017). Gel images were captured digitally (Gel Printer Plus) and saved as TIFF files. The bands on each lane were recorded as binary data (i.e., presence = 1 and absence = 0), and a binary matrix was constructed by BioNumerics software, Version 1.5 (Applied Maths, Kortrijk, Belgium). The genetic similarity coefficient among the genotypes was estimated by NTSYS-pc software, version 2.10 (Exeter Software, Setauket, New York). A dendrogram was generated for the analysis of genetic diversity based on the neighbour-joining (NJ) method.

### Detection of virulence genes by PCR

Thirteen virulence genes associated with the virulence of *A. veronii* were examined. PCR assays for the amplification of aerolysin (*aerA*), cytotoxic enterotoxin (*act*), cytotonic enterotoxins (*ast*, *alt*), lipase (*lip*), glycerophospholipid:cholesterol acyltransferase (*gcat*), serine protease (*ser*), DNase (*exu*), elastase (*ahyB*), the type III secretion system (*ascV*), the type IV secretion system (*traJ*), and haemolysin A (*hlyA*) genes and the structural gene encoding flagellin (*fla*) were performed with the template DNA of 46 *A. veronii* isolates. The conditions and the sequences of primers used for amplifying virulence genes from *A. veronii* were provided in Supplementary Table S1. PCR was performed in a 25 μl volume containing 12.5 μl 2 × PCR Master Mix (Thermo Fisher, United States), 1 μl forward primer (10 μM), 1 μl reverse primer (10 μM; synthesized by Sangon Biotech Co., Ltd., Shanghai, China), 1 μl DNA template and 9.5 μl nuclease-free water. The negative control contained all the components of the reaction mixture except DNA template, for which sterile distilled water was substituted. The reaction was performed using an Eppendorf Mastercycler nexus gradient thermoblock (Eppendorf, Germany). Subsequently, 5 μl volumes of the PCR products were analysed on a 1.5% agarose gel, and the electropherogram were visualized using a UV transilluminator (Bio-Rad, United States). Each PCR was performed in triplicate to confirm reproducibility. A dendrogram was constructed using the same method described above.

### Detection of AMR genes

A total of 22 pairs of PCR primers for seven major AMR-related elements, including aminoglycosides, β-lactams, tetracyclines, quinolones, resistance plasmids, integrons and transposons, were selected to detect the distribution of the AMR genes of 46 *A. veronii* strains isolated from aquatic systems in the Poyang Lake area. PCR amplification of extended spectrumβ-lactamase (ESBL; *blaTEM*, *blaSHV*, *blaCTX-M* and *BlaOXA*), tetracycline resistance (*tetA*, *tetB*, and *tetE*), aminoglycoside resistance [*strA-strB*, *aphAI-IAB*, *aac(3β)-IIa*, and *aac(6β)-Ib*], plasmid-mediated quinolone resistance (PMQR; *qnrA*, *qnrB*, and *qnrS*), integron (*IntI*, *IntII*), transposon (*TnpA*), and transmissible plasmid (*Qu*, *C12*, *H11*, *P12*, *F12*) genes was performed. The PCR primers and conditions were summarized in Supplementary Table S2. The negative control contained all the components of the reaction mixture except DNA template, for which sterile distilled water was substituted. PCR products were detected by electrophoresis in 2% (w/v) agarose gels. Each PCR was performed in triplicate to confirm reproducibility. A dendrogram was constructed using the same method described above.

### Determination of antimicrobial susceptibility

All 46 isolates of *A. veronii* were detected for drug susceptibility testing with the disk diffusion technique (Clinical and Laboratory Standards Institute, 2018). The plates were incubated at 28°C for 18 h under aerobic conditions. The diameter of the inhibition zone of inhibition around each disk was measured and recorded. Each bacterial strain was classified as resistant (R), intermediately resistant (I), or susceptible (S) according to the guidelines of the Clinical and Laboratory Standards Institute (2018). In all tests, the standard strain *Escherichia coli* ATCC 25922 was used as a control. The following antibiotics, which were classified into eight different categories according to their chemical structure, were tested: aminoglycosides: amikacin 30 μg (AK); neomycin 30 μg (N) and gentamycin 10 μg (GM); β-lactams: ceftriaxone 30 μg (CTRX) and ampicillin 10 μg (AMP); quinolones: enrofloxacin 5 μg (ENR); norfloxacin 10 μg (NOR) and ofloxacin 5 μg (OFX); chloramphenicols: chloramphenicol 30 μg (C) and florfenicol 30 μg (FFC); macrolides: midecamycin 30 μg (MID); erythromycin 15 μg (ERY); polymyxins: polymyxin-B 300 U (PB); tetracyclines: tetracycline 30 μg (TET); doxycycline 30 μg (DOX) and sulfonamides: trimethoprim/sulfamethoxazole 1.25 μg and 23.75 μg (SXT).

### Statistical analysis

Statistical analysis of the virulence genes and the distribution and clustering of AMR genes were performed using Excel 2007 and the Statistical Package for the Social Sciences (SPSS), version 19.0.

## Results

### Eric-PCR

**Figure 2:**
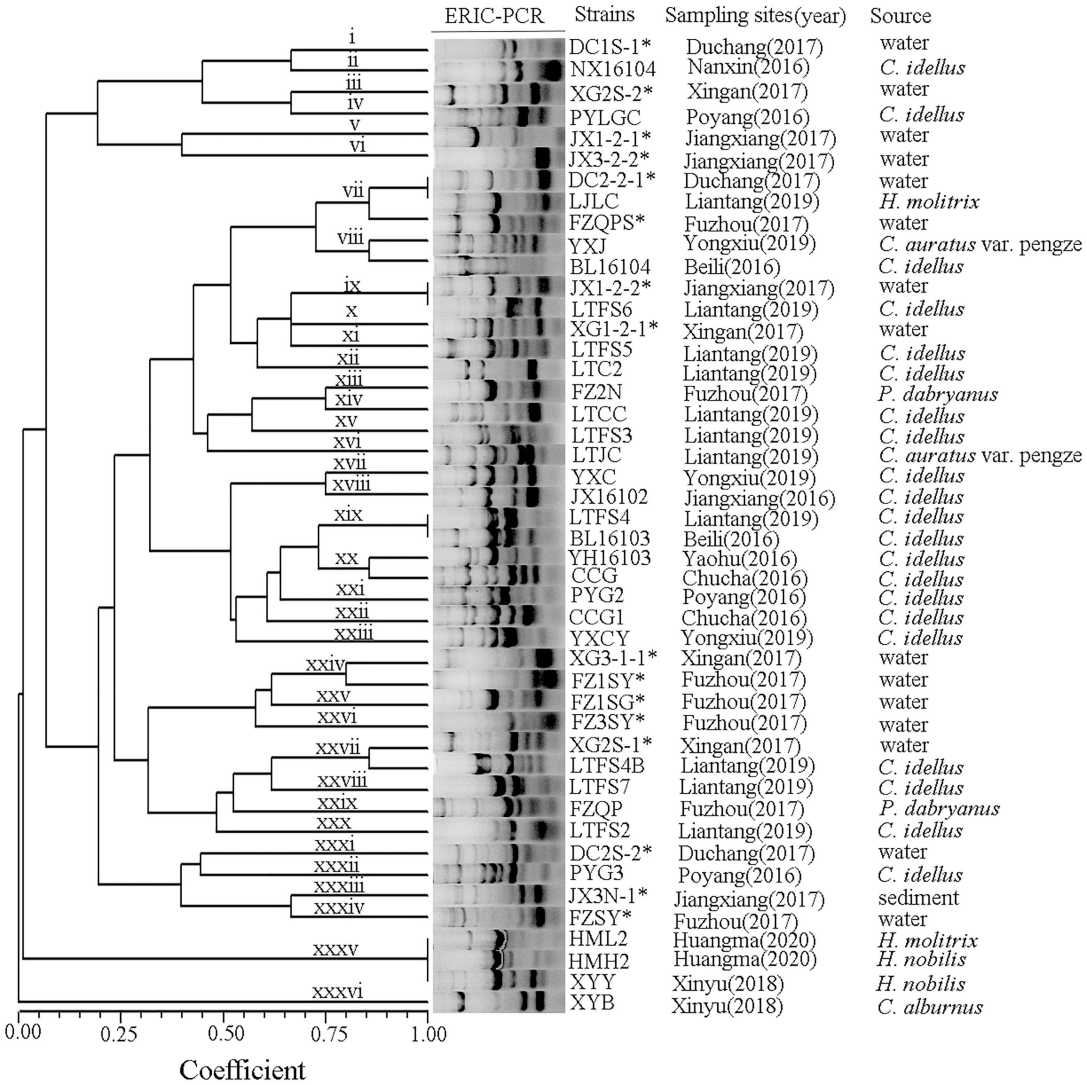
Dendrogram showing the ERIC profiles of the *A. veronii* isolates used in this study. “*“indicated the strains isolated from pond water or sediment (the same below).

ERIC primers produced between 4 and 10 bands per reaction with sizes ranging from 0.1 to 7.0 kb (Figure 2). *A. veronii* isolates from the Poyang Lake areas produced forty-six ERIC patterns, and all isolates yielded products with the primers used. The *A. veronii* strains exhibited 36 genotypes (i ~ xxxvi) with a genetic similarity coefficient of 80%. Isolates from the same location and the same year were genetically heterogeneous.

### PCR amplification of virulence genes

**Figure 3:**
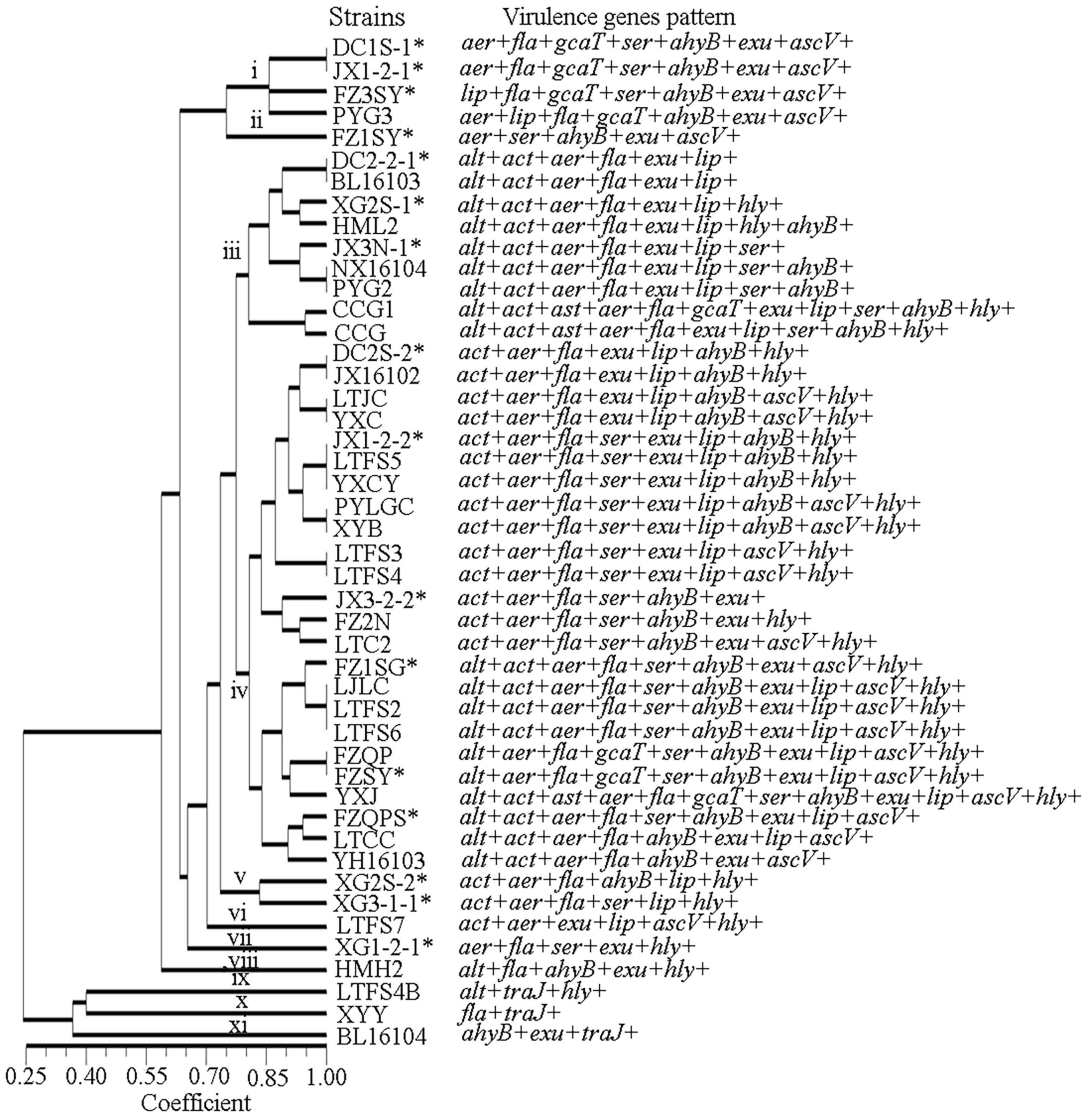
The virulence genes pattern of the *A. veronii* isolates used in this study.

The fewest virulence-related genes were found in isolate XYY, which had only 2 such genes, *fla* and *traJ*. Isolate YXJ was found to possess 12 virulence-related genes, making it the isolate with the most virulence-related genes. The forty-six isolates with a genetic similarity coefficient of 80% were divided into 11 virulence-related gene patterns (Figure 3). Patterns iii and iv were the predominant virulence-related gene patterns, found in 9 and 21 isolates, respectively. The pattern iii isolates carried the genes *alt*, *act*, *aer*, *fla*, *exu* and *lip* genes, and the pattern iv isolates contained the *act*, *aer*, *fla*, *exu*, *lip*, *ahyB* and *hly*.

All 13 of the virulence genes tested for were detected. The most frequently detected genes were *exu* (93%, 43/46), *fla* (91%, 42/46), *aer* (89%, 41/46), *ahyB* (76%, 35/46), *act* (74%, 34/46), *lip* (72%, 33/46), *ser* (63%, 28/46), *asc* (50%, 23/46), and *alt* (46%, 21/46).

**Figure 4:**
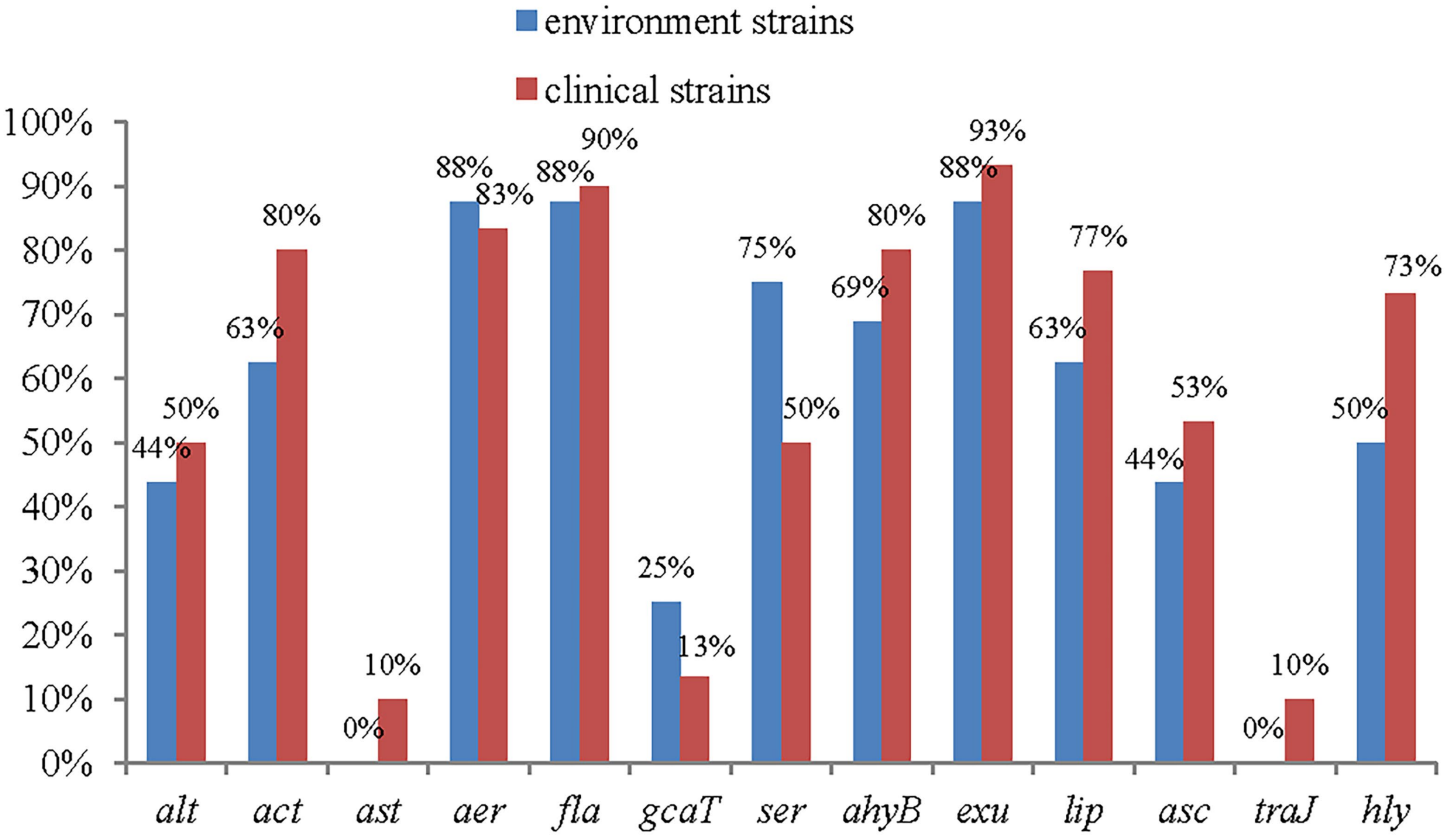
The distribution of virulence genes in clinical strains and environment strains of *A. veronii*.

The genes *Ast* and *Tra* were present at the lowest frequencies, at 7% each (3/46). Among the 13 detected virulence genes, 10 had higher frequencies among clinical strains than among environmental strains, and only 2 had lower frequencies among clinical strains than among environmental strains. Significant differences were found among the *act*, *lip* and *hly* genes. The *ast* and *traJ* genes were detected only in clinical isolates (Figure 4).

### Patterns of AMR genes

**Figure 5:**
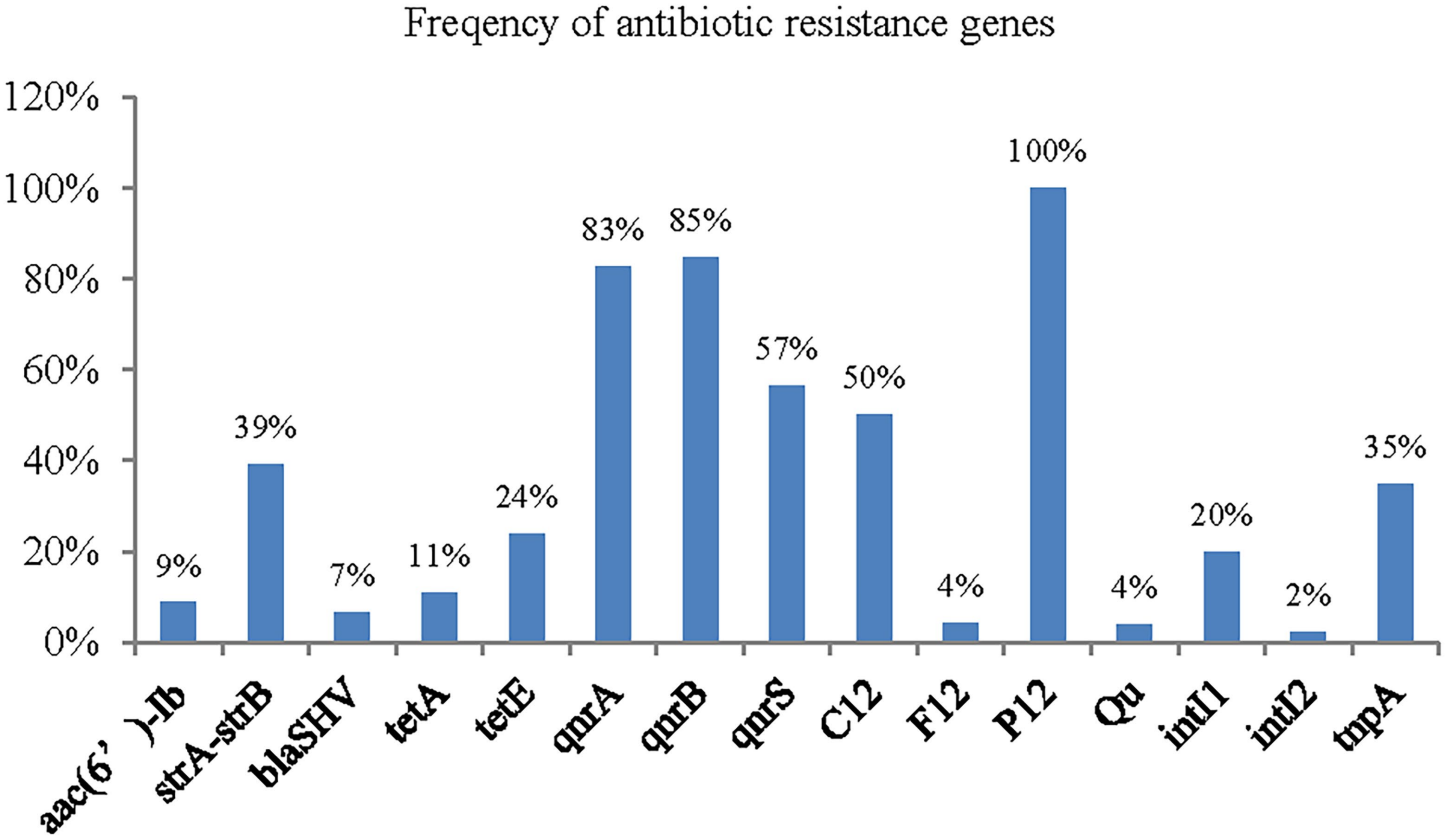
The frequency of antimicrobial resistance genes of *A. veronii.*

A total of 15 AMR genes were detected, *aphAI-IAB*, *aac(3β)-IIa*, *bla_TEM_*, *bla_CTM-M_*, *Bla_OXA_*, and *tetB* and the plasmid gene *H11* were not detected (Figure 5). The results showed that quinolone drug resistance genes were ubiquitous aross the *A. veronii* strains, with 82% (38/46) strains containing *qnrA*, 84% (39/46) containing *qnrB*, and 56% (26/46) containing *qnrS*. Two of the four investigated aminoglycoside resistance genes were detected: *strA-strB*, with a frequency of 39% (18/46), and *aac*(6′)*-Ib*, with a frequency of 8% (4/46).

Only a small number of β-lactam resistance genes and tetracycline resistance genes were detected among the 46 strains of *A. veronii*. Regarding the β-lactam resistance genes, *bla_SHV_* was detected in only 6% (3/46) of the strains, and no other β-lactam genes were detected. Two tetracycline resistance genes were detected: *TetA*, with a frequency of 10% (5/46), and *TetE*, with a frequency of 23% (11/46).

The plasmid *P12* gene was widely distributed among the *A. veronii* strains, with a frequency of 100%. Half of the strains contained the plasmid gene *C12* plasmid gene. A few strains contained the plasmid genes *F12* plasmid (4%, 2/46) and Qu (4%, 2/46). The plasmid gene H11 was not detected. In addition, the frequency of the class 1 integron gene *intI* was 19% (9/46), and that of the class 2 integron gene *intII* was 2% (1/46). The frequency of the transposon gene *tnpA* was 34% (16/46).

**Figure 6:**
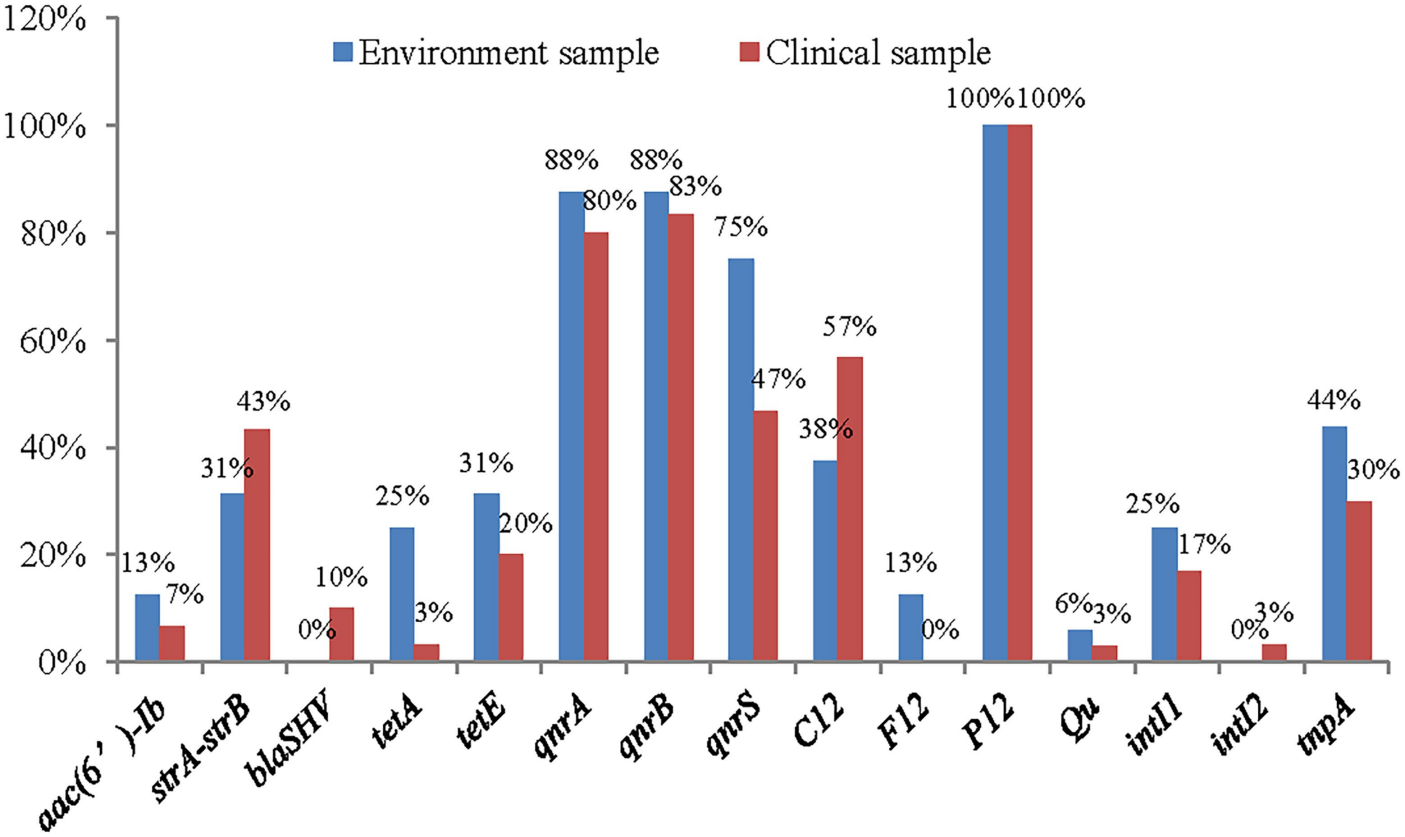
The distribution of antimicrobial resistance genes in clinical strains and environment strains of *A. veronii.*

Of the 15 genes detected, 10 had higher frequencies among environmental strains, and only 4 had higher frequencies in clinical strains (Figure 6).

**Figure 7:**
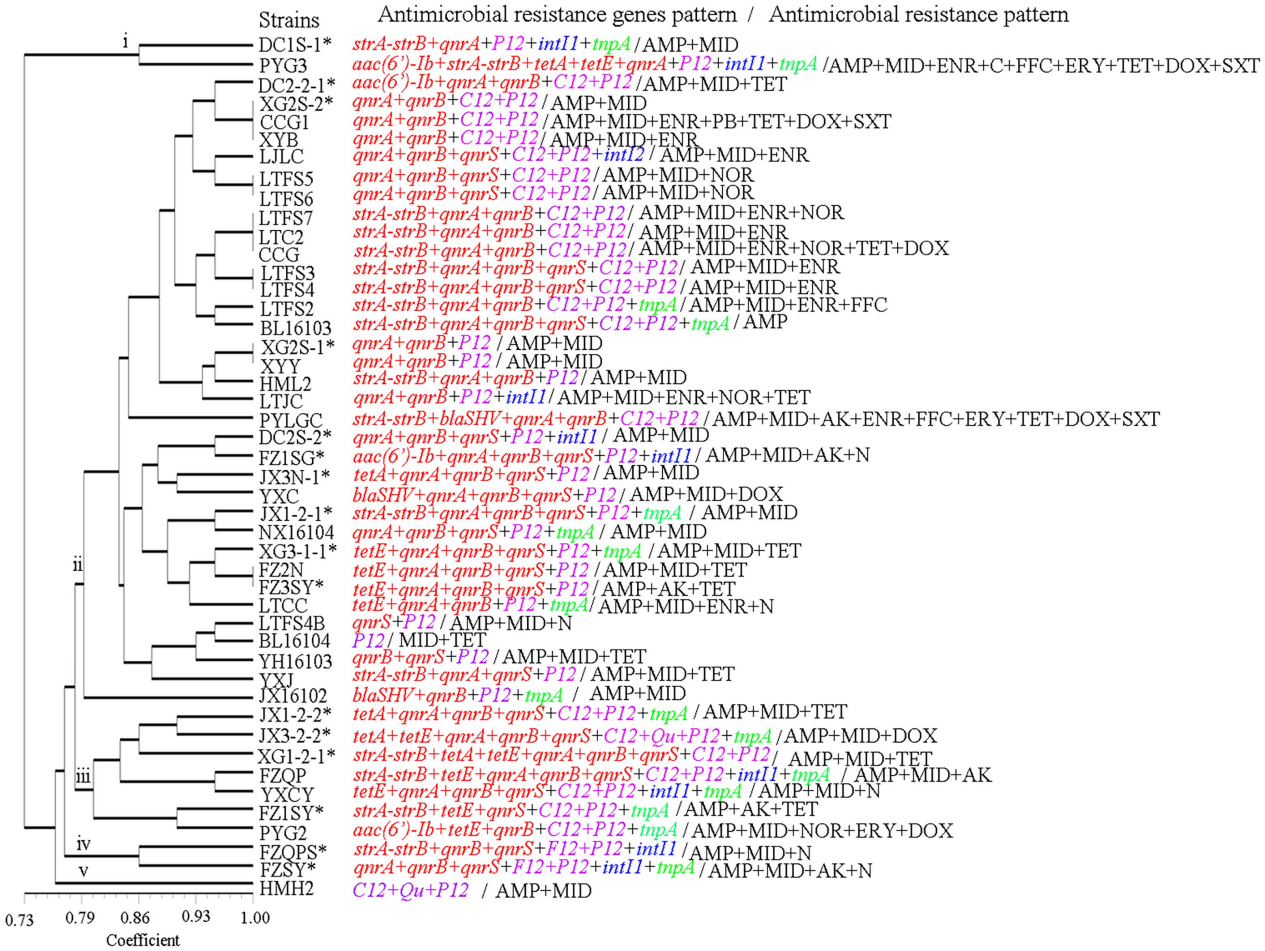
The antimicrobial resistance genes and antimicrobial resistance pattern of the *A. veronii* isolates used in this study. AK, amikacin; N, neomycin; GM, gentamycin; CTRX; ceftriaxone; AMP, ampicillin; NOR, norfloxacin; OFX, ofloxacin; ENR, enrofloxacin; C, chloramphenicol; FFC, florfenicol; MID, midecamycin; ERY, erythromycin; PB, polymyxin-B; TET, tetracycline; DOX, doxycycline; SXT, trimethoprim/sulfamethoxazole.

*Via* AMR gene genotyping, the 46 isolates with a genetic similarity coefficient of 80%, were found to exhibit 5 patterns of antibiotic resistance genes. Pattern ii was the main antibiotic resistance gene pattern, found in 34 isolates (Figure 7).

### Antimicrobial susceptibility

The antimicrobial susceptibility results for the 46 strains of *A. veronii* showed that almost all the strains were resistant to ampicillin and midecamycin. Twenty-six percent of the strains were resistant to enrofloxacin, 22% were resistant to tetracycline, 13% were resistant to neomycin and 11% were resistant to amikacin, norfloxacin and doxycycline. Three strains were partially or fully resistant to gentamycin, chloramphenicol, florfenicol, polymyxin-B and trimethoprim/sulfamethoxazole. No strains were resistant to both ceftriaxone and ofloxacin.

**Figure 8:**
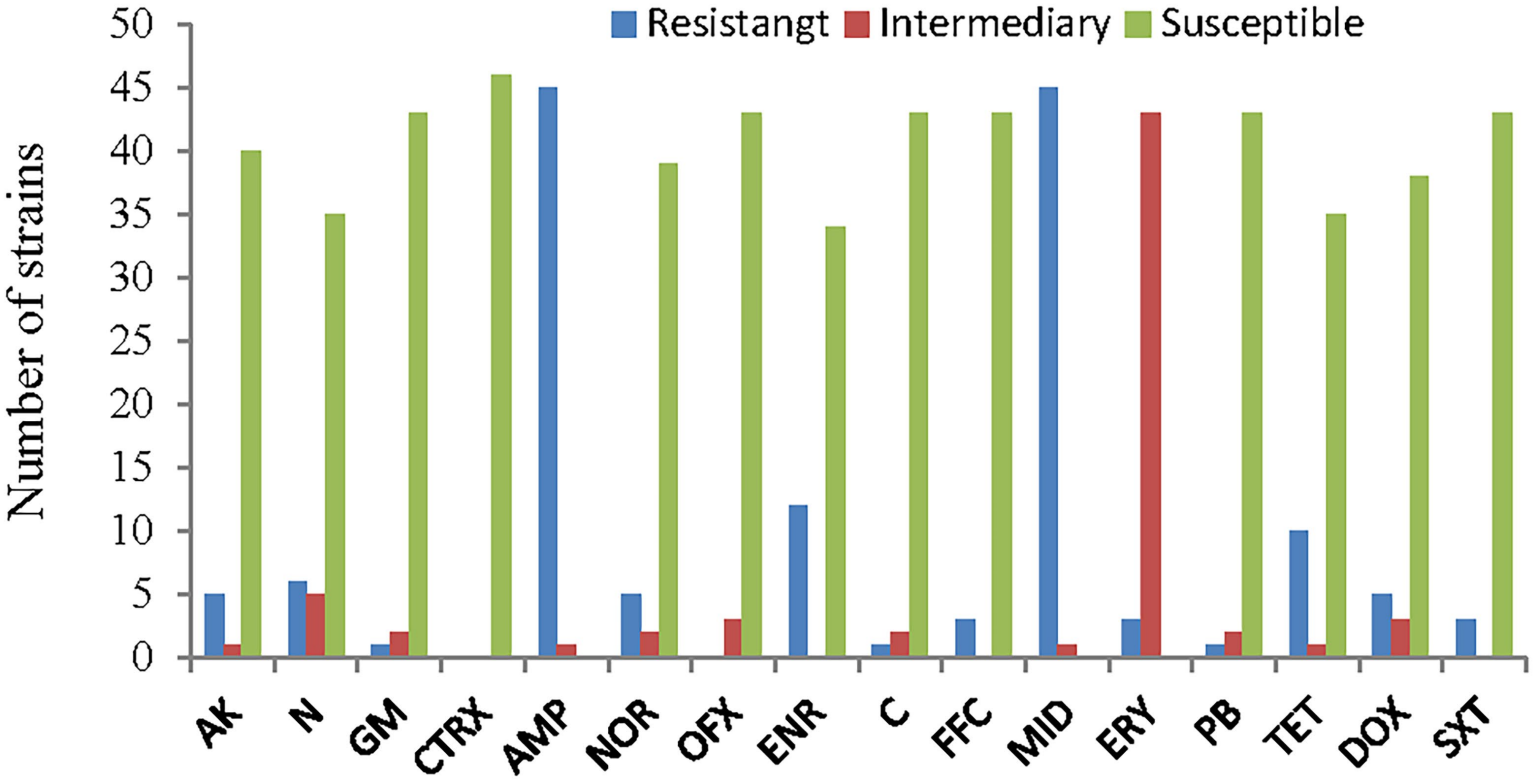
Antimicrobial susceptibility of the *A. veronii* isolates used in this study. AK, amikacin; N, neomycin; GM, gentamycin; CTRX; ceftriaxone; AMP, ampicillin; NOR, norfloxacin; OFX, ofloxacin; ENR, enrofloxacin; C, chloramphenicol; FFC, florfenicol; MID, midecamycin; ERY, erythromycin; PB, polymyxin-B; TET, tetracycline; DOX, doxycycline; SXT, trimethoprim/sulfamethoxazole.

The most resistant strains were PYG3 and PYLGC, which were each resistant to 9 kinds of antibiotics, followed by CCG1 and CCG, which were resistant to 7 and 6 kinds of antibiotics, respectively. The least resistant strain was BL16103, which showed resistance to only one drug. The remaining strains were resistant to 2, 3 or 4 drugs (Figures 7, 8; Supplementary Table S3).

## Discussion

ERIC-PCR is one of the most popular methods for genotyping *Aeromonas* because it is easy to carry out, does not require any expensive equipment and is highly reproducible (Fernández-Bravo and Figureueras, 2020). Genetic analysis of *A. veronii* performed *via* ERIC-PCR has revealed the epidemiological relationships of various strains and the genetic diversity of this species (Ye et al., 2012). In this study, the population of *A. veronii* was genetically heterogeneous, with the 46 strains producing 36 ERIC-PCR types and lacking dominant clones. Some strains from the same source produced different ERIC types: for example, FZQPS, FZ2N, FZQP, and FZSY were isolated from the same aquatic farm but had different ERIC types. A similar result was observed for strains DC2-2-1 and DC2S-2. In contrast, some strains from different sources sampled in different years exhibited similar genotypes, such as the type vii strains DC2-2-1 and LJLC and the type xix strains LTFS4 and BL16103. The high diversity of *A. veronii* poses a problem for disease control. Different types of bacteria have different properties and environmental adaptabilities and correspondingly can infect a wide range of hosts, which is in line with the prevalence of *A. veronii* infection in a variety of aquatic animals in southern China in recent years (Ran et al., 2018; Gao et al., 2020; Wang et al., 2021).

We examined the numbers and types of virulence genes that are essential for *Aeromonas* infection. Aravena-Román et al. (2014) expounded, in general, that pathogens should possess the virulence genes necessary to gain entry, adhere, and colonize host tissue, causing damage, while evading host defence mechanisms and, in some cases, spreading, leading to systemic infection. In this study, virulence genes related to entry (*gcaT, exu, ser, lip, ascV, TraJ*), adherence (*fla*), colonization (*ahy*, *act*, *alt*, *ast*, *hly*, *aer*) and other processes were selected for survey.

The genes *exu*, *fla* and *aer* were the most frequently observed genes, with frequencies of 93, 91 and 89%, respectively. These results are consistent with those of Khor et al. (2015), who found that the frequencies of the genes *exu*, *fla* and *aer* were 95, 86 and 93%, respectively, in 44 strains of *A. veronii* isolated from freshwater lakes in Malaysia. In addition, the results are consistent with those of Nawaz et al. (2010), who found that among 81 strains of *A. veronii* isolated from farm-raised catfish, 80 and 96% contained the genes *fla* and *aer*, respectively. The gene *aerA* codes for aerolysin, and had been identified as the key virulence factor of *A. veronii* in southern China (Cirauqui Diaz et al., 2017; Ran et al., 2018). The high percentages of isolates positive for these genes highlight the relevance of these genes for the maintenance of *Aeromonas* spp. in the host.

Haemolysin, which is encoded by haemolysin genes (such as *hlyA*), associated with aerolysin (*aerA*) and participates in virulence by inducing incomplete erythrocyte lysis (a-haemolysins) or the complete destruction of erythrocytes (β-haemolysins; Tomás, 2012). In this study, the frequency of *hlyA* was 65%, consistent with the frequency of 65% reported by Dahanayake et al. (2020), among *A. veronii* strains isolated from *Tegillarca granosa* in Korea.

Three enterotoxin genes (*act*, *ast* and *alt*) related to public health were detected in this study. Any one of these three enterotoxins can lead to severe diarrhoea. The presence of all three enterotoxin genes in an *Aeromonas* isolate could be devastating to patients (Chopra et al., 2009). In this study, 2 out of the 46 strains contained all three of these genes. These three enterotoxin genes are related to human diseases and cause diarrhoea, enteritis and other conditions. Among them, *ast* and *alt* are generally abundant in *A. hydrophila*. For example, Aravena-Román et al. (2014) reported that among *A. hydrophila* strains, 87 and 91% of clinical strains carried *alt* and *ast*, respectively, and 83 and 100% of environmental strains carried *alt* and *ast*, respectively. Similarly, Zhou et al. (2019) reported that 66% of *A. hydrophila* strains carried the gene *alt* and that 100% carried the gene *ast*. In this study, *ast* and *alt* were detected in *A. veronii*, which does not exclude the possibility that virulence genes may be transferred between *A. hydrophila* and *A. veronii* through mobile genetic elements (MGEs), such as plasmids, insertion sequence (IS) elements, transposons, genomic/pathogenicity islands, and integron-associated gene cassettes, between *A. hydrophila* and *A. veronii* (Piotrowska and Popowska, 2015).

The TTSS is an important virulence factor in *Aeromonas* strains (Reyes-Rodríguez et al., 2019). In this study, the frequency of the gene *ascV*, which encodes a component of the TTSS, was found to be 50%, which is slightly lower than the 66% reported by Reyes-Rodríguez et al. (2019) and higher than the 31% reported by Aravena-Román et al. (2014). The frequency of *ascV* was higher in clinical strains (53%) than that in environmental strains (45%) in this study, which is consistent with the findings of Vilches et al. (2004) and Reyes-Rodríguez et al. (2019). However, Aravena-Román et al. (2014) observed the opposite pattern, reporting that 50% (4 of 8) of environmental strains carried *ascV*, while only 23% (6 to 26) of clinical strains had this gene. Based on these results, it appears that the TTSS might not be a key virulence factor of *A. veronii* and that the distribution patterns of the TTSS genes are strain- and source-dependent (Aravena-Román et al., 2014).

TFSSs are deployed by many bacterial species to deliver DNA, protein, or other macromolecules to bacterial or eukaryotic cell targets or even to the environment (Wallden et al., 2010; Li et al., 2019; Costa et al., 2021). TFSSs have not been extensively studied in *A. veronii* (Matys et al., 2020). Rangrez et al. (2010) reported a case of a TFSS encoded by a plasmid. In this study, only 2 clinical strains were found to carry the investigated TFSS gene. However, because TFSSs can mediate horizontal gene transfer (Wallden et al., 2010), which greatly facilitates adaptation to environmental changes and is the basis for the spread of virulence and even drug resistance among bacteria, the distribution of TFSSs in *A. veronii* warrants continued attention.

The frequencies of *lip*, *ahyB*, *ser* and *gcaT,* genes that encode extracellular enzymes, were 72, 76, 63 and 17%, respectively, in this study. These findings differ to some extent from those of other reports. For example, Dahanayake et al. (2019) reported that the frequencies of *lip*, *ahyB*, and *gcaT* were all 20%, and that the frequency of *ser* was 0. Nawaz et al. (2010) reported frequencies of *ser*, *gcaT* and *lip* of 82, 78 and 85%, respectively. Zhou et al. (2019) found 8.3% positivity for the gene *lip* among 36 *A. veronii* strains.

The *A. veronii* strains isolated from the Poyang Lake area contained multiple virulence factors, and the main types were *alt + act + aer + fla + exu + lip* (9/46) and *act + aer + fla + exu + lip +  ahyB + hly* (24/46). These results are similar to those of Khor et al. (2015) and Roges et al. (2020), which indicates that the virulence of *A. veronii* is a synergistic effect of multiple virulence factors. Notably, 78% (36/46) of the strains contained both *act* and *aer*. According to Nawaz et al. (2010), bacteria containing the genes *act* and *aer* are virulent strains, which indicates that the virulent strains of *A. veronii* are dominant in the Poyang Lake area.

The results of AMR gene detection by PCR amplification showed that AMR genes were widely distributed among *A. veronii* strains isolated from the Poyang Lake area. The percentage of strains containing two or more AMR genes was 98% (45/46), and 76% of the strains contained five or more AMR genes, which to some extent reflected the law of “survival of the fittest” for bacteria (Munita and Arias, 2016). That is, widespread antibiotic contamination in the natural environment has placed selective pressure on *A. veronii* such that populations with few or no AMR genes have been unable to persist in the natural environment. Only individuals with multidrug resistance genes have been able to survive in the presence of the antibiotic components in the environment that are derived from human activities. The results also showed that there has been antibiotic abuse in the aquaculture system in the Poyang Lake area: the majority of individuals in the group carried five or more drug resistance genes, indicating the existence of selection for antibiotic resistance.

In this study, an abundance of PMQR genes was observed among the tested *A. veronii* strains. Among these genes, *qnrB* (85%) was the most prevalent among the isolates, followed by *qnrA* (83%), and their abundances were higher than those in previous reports (Verner-Jeffreys et al., 2009; Hossain et al., 2018). The lowest abundance of the *qnr* genes was 57% in this study. There may be geographical differences associated with the abundance of these genes. Quinone drugs, such as enrofloxacin, have been authorized for use in Chinese aquaculture, and are used in large quantities in China (Liu et al., 2017). The increased use of quinolones has led to a rise in bacterial resistance (Kolár et al., 2001).

The tetracycline resistance genes *tetE* and *tetA* were detected in 24 and 11% of the isolates, respectively, whereas *tetB* was not detected in any isolate. The genes *tetA* and *tetE* are the most common tetracycline resistance determinants of *Aeromonas* spp. (Hossain et al., 2018). In this study, two *tet* genes were detected in the *A. veronii* strains isolated from the Poyang Lake area, occasionally in the same strain (strains JX3-2-2 and XG1-2-1). This result indicated that resistance to tetracycline was high among strains in the Poyang Lake area, which was also suggested by the drug susceptibility test results of this study. For example, a total of 22% of the strains analysed in this study were resistant to tetracycline. The reason may be related to the fact that tetracycline has been approved for and is frequently used in veterinary medicine in China. A large number of tetracycline drugs have been used for the prevention and treatment of aquatic diseases, resulting in *A. veronii* developing resistance to tetracycline. Some strains in which *tet* genes were not detected also presented resistance phenotypes, indicating that there might be other genes conferring resistance to tetracycline that were not detected, possibilities include the *tet* (34) gene, which is widely distributed in *A. veronii* (Tekedar et al., 2020).

In this study, two aminoglycoside resistance genes, *strA-strB* and *aac(6β)-Ib*, were detected in 39 and 9%, respectively, of *A. veronii* strains. This result is consistent with a previous report in which 5% of *A. veronii* strains contained the gene *aac(6β)-Ib* (Deng et al., 2014). In contrast, Dahanayake et al. (2019) and Hossain et al. (2018) reported that the frequency of *aac(6β)-Ib* in *A. veronii* reached 67 and 56%, respectively. These differences among studies show that there are regional differences in the distribution of aminoglycoside resistance genes, as drug use policies vary among countries, differences in selective pressure exist, leading to differences in the distribution of resistance genes. In China, only one aminoglycoside drug, neomycin sulfate, has been approved for use in aquaculture, which might explain the lower resistance to aminoglycosides than to other antibiotic drugs among the genotypes and phenotypes of *A. veronii* in the Poyang Lake area (Committee of Chinese Veterinary Pharmacopoeia, 2021). Among the surveyed ESBL genes, only *bla_SHV_* was observed, detected in 7% of the isolates. *Bla_TEM_*, *Bla_CTX-M_* and *Bla_OXA_* were not detected in this study. These results slightly differ from those of previous studies. For example, Dahanayake et al. (2019) detected the *bla_SHV_* and *bla_CTX_* in 33 and 20%, respectively, of *A. veronii* strains isolated from marketed cockles in Korea. Fauzi et al. (2021) reported that 47% of *A. veronii* strains isolated from fish carried the gene *Bla_TEM_*, and that 11% of strains carried *Bla_SHV_*. These differing results show that there are also geographic differences in the distribution of ESBL genes among *A. veronii* strains.

Integrons, transposons and mobile plasmids are the most frequent genetic elements involved in the horizontal spread of resistance genes and have facilitated the global antibiotic resistance crisis. They are a potential force in the adaptation and rapid evolution of bacteria (Alvarado et al., 2012; Gillings, 2014; Khademi et al., 2021). Some researchers have analysed the genomes of *A. veronii* strains and, shown that strains containing integrons, transposons and plasmids have more putative AMR genes than other *A. veronii* strains (Tekedar et al., 2019). In this study, 20% of the *A. veronii* strains isolated from the Poyang Lake area carried class 1 integrons, this finding is consistent with previous reports, showing that 21% ~ 31% of *Aeromonas* spp. strains isolated from aquatic environments were positive for class 1 integrons (Carnelli et al., 2017; Dhanapala et al., 2021). It seems that the frequency of class 1 integrons in *Aeromonas* spp., including *A. veronii* strains isolated from polluted environments, is high. Therefore, class 1 integrons of *A. veronii* can be used as indicators of pollution originating from human activity, and the antibiotic resistance phenotypes can be comprehensively evaluated by detecting the distribution frequency of class 1 integrons in *A. veronii* strains. In addition, slass 2 integrons that confer antibiotic resistance in clinical contexts have been described. The frequency of class 2 integrons in environmental microorganisms is low. This study confirms the existence of class 2 integrons in *A. veronii*. Similar to class 1 integrons, class 2 integrons evolve under selective pressure imposed by human activity and continue to accumulate new gene cassettes relevant to resistance, pathogenicity and virulence (Gillings, 2014). Thus, the monitoring of class 2 integrons should be increased to reveal the mechanisms of bacterial resistance, pathogenicity and virulence.

Plasmids can mediate horizontal gene transfer of antibiotic resistance, virulence genes, and other adaptive factors across bacterial populations (Lopatkin et al., 2017; Redondo-Salvo et al., 2020). *Aeromonas* spp. carry a variety of plasmids. Some plasmids showed template or host genes acquired from *Pseudomonas monteilii*, *Salmonella enterica*, *Escherichia coli* or other species (Nwaiwu and Aduba, 2020). In this study, plasmid gene fragments of *Escherichia*, *Salmonella* and *Yersinia* were detected in *A. veronii*, indicating that plasmids of these bacteria could be transferred to *A. veronii*. Among the plasmids detected in *A. veronii*, the *Escherichia coli* resistance plasmid R64 was the most common, with a transfer frequency reaching 100%. Previous studies have reported that both *Escherichia* and *Aeromonas* are abundant in water, with similar ecological niches (Araujo et al., 1989; Carnelli et al., 2017; Solaiman and Micallef, 2021). By employing metagenomics technology, researchers have revealed the existence of “habitat-specific gene pools,” suggesting that transmission is more frequent among species occupying the same ecological niche (Redondo-Salvo et al., 2020). The results of this study are consistent with these findings. Of course, the presence of AMR genes in plasmids may not necessarily translate to strong phenotypic expression (Nwaiwu and Aduba, 2020). However, plasmids are a transferable AMR gene pool, and the threat they pose to public health should be noted. Continuous monitoring can help us better understand the impact of plasmids on the evolution and persistence of bacterial species (Redondo-Salvo et al., 2020).

Transposons are important for bacterial evolution and adaptation because they play a central role in disseminating resistance to antibiotics in gram-negative bacteria. They are often associated with multidrug resistance plasmids in *Aeromonas* (Del Castillo et al., 2013). In this study, transposons were found in 35% of the *A. veronii* strains; this frequency is higher than the 14% reported in *Aeromonas* spp. isolated from aquatic environments of Switzerland (Carnelli et al., 2017). A possible reason for this differences is that the strains studied in this paper originated from aquaculture systems, while the strains in the study of Carnelli et al. (2017) originated from four water bodies, including unpolluted waters, this differences suggests a positive correlation between the frequency of transposons in *A. veronii* and the human use of antibiotics. Previous reports have shown that transposons are important for bacterial adaptation and evolution in marine ecosystems (Dahlberg and Hermansson, 1995). This study indicated that transposons may also play important roles in evolution and adaptability in freshwater ecosystems.

There are four varieties of natural antibiotics and two classes of synthetic antibiotics approved by the Ministry of Agriculture and Rural Affairs of the People’s Republic of China for production and use in aquaculture: neomycin, doxycycline, florfenicol, thiamphenicol, sulfonamides, and quinolones (Committee of Chinese Veterinary Pharmacopoeia, 2021). In this study, these approved antibiotics and other common antibiotics were selected for drug sensitivity testing, and the results showed that all *A. veronii* isolates were highly ampicillin resistant. This finding is likely due to high intrinsic β-lactam resistance (Tayler et al., 2010). Furthermore, high resistance to quinolones, tetracyclines and aminoglycosides was detected, which might be due to the extensive consumption of such antimicrobials in aquaculture systems in the Poyang Lake area. Additionally, although the phenotypic resistance of most strains was consistent with the AMR genotype in our study, some strains with drug resistance genes, such as strains BL16106 and CCG1, did not show phenotypic resistance. This result is consistent with previous studies identifying discrepancies between phenotypic and genotypic traits; these studies indicate that these discrepancies might be the result of resistance gene silencing or mutation (Carnelli et al., 2017; Dahanayake et al., 2019; Baunoch et al., 2021). These observations indicate that one cannot predict the bacterial antibiotic response only by detecting AMR genes in *A. veronii*.

## Conclusion

This study showed that *A. veronii* in the Poyang Lake area has high genetic diversity and no dominant clones. There was great variation in virulence genes and AMR gene occurrence, possession and distribution among the *A. veronii* isolates. The frequency of virulence genes among clinical strains was higher than that among environmental strains, while the distribution of AMR genes showed the opposite pattern. There were no regional or temporal differences in the distribution of virulence genes or AMR genes among the *A. veronii* strains, indicating that the *A. veronii* strains in the Poyang Lake area have strong adaptability to the environment and that gene exchange is actively occurring among strains in this area, which may be due to the mutual spread of strains in the Poyang Lake area due to floods or the circulation of fry. Some strains were resistant to a variety of traditionally effective drugs for *Aeromonas,* such as quinolones, tetracyclines and aminoglycosides, indicating that selective resistance of bacteria caused by the abuse of antibiotics has emerged in the Poyang Lake area. These results emphasize the importance of monitoring *A. veronii* in aquatic systems to help prevent and control any future public health crises.

## Data availability statement

The original contributions presented in the study are included in the article/Supplementary material, further inquiries can be directed to the corresponding author/s.

## Author contributions

XX, CW, and HF conceptualized the project and designed the experiments. GW and JH collected samples. ZZ and YR collected samples and performed the experiments. LL assisted in experiments. XX collected samples, analyzed the data and wrote the manuscript. All authors approved the final version of the manuscript.

## Funding

This study was supported financially by the earmarked fund for Jiangxi Agriculture Research System (no. JXARS-03); the Key research and development program of Jiangxi Province (no. 20202BBFL63024); the Key Laboratory of Fishery Drug Development of Ministry of Agriculture and Rural Affairs, and the Key Laboratory of Aquatic Animal Immune Technology of Guangdong Province (no. 2019-05).

## Conflict of interest

The authors declare that the research was conducted in the absence of any commercial or financial relationships that could be construed as a potential conflict of interest.

## Publisher’s note

All claims expressed in this article are solely those of the authors and do not necessarily represent those of their affiliated organizations, or those of the publisher, the editors and the reviewers. Any product that may be evaluated in this article, or claim that may be made by its manufacturer, is not guaranteed or endorsed by the publisher.
