# Supplementary material for: Prevalence and genetic diversity of Aeromonas veronii isolated from aquaculture systems in the Poyang Lake area, China
Source: Front Microbiol. 2022 Dec 12;13:1042007. doi: 10.3389/fmicb.2022.1042007 (PMC9791064; doi:10.3389/fmicb.2022.1042007)
Supplement: Supplementary file 1 [file Table_1.DOCX]

Supplementary table 1 Primers and PCR conditions*^a^* for virulent factors.

| Gene | Virulent factors | Primer sequence(5' to 3') | Annealing temperature (ºC) | Amplicon  size (bp) | Reference |
| --- | --- | --- | --- | --- | --- |
| *hlyA* | Hemolysin A | F:GGCCGGTGGCCCGAAGATACGGG | 62 | 597 | Wong et al.  1998 |
|  |  | R:GGCGGCGCCGGACGAGACGGG |  |  |  |
| *alt* | Heat-labile cytotonic enterotoxin | F:TGACCCAGTCCTGGCACGGC | 63 | 442 | Nawaz et al. 2010 |
|  |  | R:GGTGATCGATCACCACCAGC |  |  |  |
| *act* | Cytotoxic enterotoxin | F:AGAAGGTGACCACCACCAAGAACA | 64 | 232 |  |
|  |  | R:AACTGACATCGGCCTTGAACTC |  |  |  |
| *ast* | Heat-stable cytotonic enterotoxin | F:TCTCCATGCTTCCCTTCCACT | 63 | 331 |  |
|  |  | R:GTGTAGGGATTGAAGAAGCCG |  |  |  |
| *aerA* | Aerolysin | F:CCTATGGCCTGAGCGAGAAG | 63 | 431 |  |
|  |  | R:CCAGTTCCAGTCCCACCACT |  |  |  |
| *fla* | Flagella | F:TCCAACCGTYTGACCTC | 55 | 608 |  |
|  |  | R:GMYTGGTTGCGRATGGT |  |  |  |
| *GcaT* | Glycerophospholipid-cholesterol acyltransferase | F:CTCCTGGAATCCCAAGTATCAG | 65 | 237 |  |
|  |  | R:GGCAGGTTGAACAGCAGTATCT |  |  |  |
| *ser* | Serine protease | F:CACCGAAGTATTGGGTCAGG | 57 | 350 |  |
|  |  | R:GGCTCATGCGTAACTCTGGT |  |  |  |
| *ahyB* | Elastase | F:ACACGGTCAAGGAGATCAAC | 57 | 513 |  |
|  |  | R:CGCTGGTGTTGGCCAGCAGG |  |  |  |
| *exu* | DNase | F:RGACATGCACAACCTCTTCC | 62 | 323 |  |
|  |  | R:GATTGGTATTGCCYTGCAAS |  |  |  |
| *lip* | Lipase | F:CAYCTGGTKCCGCTCAAG | 62 | 382 | Sen and Rodgers 2004 |
|  |  | R:GTRCCGAACCAGTCGGAGAA |  |  |  |
| *ascV* | Type III Secretion System | F:AGCAGATGAGTATCGACGG | 58 | 891 | Carvalho-Castro et al. 2010 |
|  |  | R:AGGCAT TCTCCTGTACCAG |  |  |  |
| *traJ* | Type IV Secretion System | F：GCCGGATCCATGTTCCGAGAAATATT | 63 | 1043 | Rangrez et al. 2010 |
|  |  | R:GCCAAGCTTACTTAAATTGATTCACTCAGC |  |  |  |

*^a^* PCR thermocycle conditions for each reaction is as follows, initial denaturation of 94 ºC for 5 min followed by a total of 35 cycles of amplification. Each cycle consisted of 94 ºC denaturation for 30 s, annealing for 50 s and 72 ºC extension for 1 min, and a final extension for 7 min at 72 ºC.

**References**

Carvalho-Castro, G.A., Lopes, C.O., Leal, C.A.G., Cardoso, P.G., Leite, R.C., Figueiredo, H.C.P., 2010. Detection of type III secretion system genes in *Aeromonas hydrophila* and their relationship with virulence in Nile tilapia. Vet. Microbiol. 144, 371-376. https://doi.org/10.1016/j.vetmic.2010.01.021.

Nawaz, M., Khan, S.A., Khan, A.A., Sung, K., Tran, Q., Kerdahi, K., Steele, R., 2010. Detection and characterization of virulence genes and integrons in *Aeromonas veronii* isolated from catfish. Food microbiol. 27, 327–331. https://doi.org/10.1016/j.fm.2009.11.007.

Rangrez, A.Y., Abajy, M.Y., Keller, W., Shouche, Y., Grohmann, E., 2010. Biochemical characterization of three putative ATPases from a new type IV secretion system of Aeromonas veronii plasmid pAC3249A. BMC biochem. 11, 10. https://doi.org/10.1186/1471-2091-11-10.

Sen, K., Rodgers, M., 2004. Distribution of six virulence factors in *Aeromonas* species isolated from US drinking water utilities: a PCR identification. J Appl. Microbiol. 97, 1077–1086. <https://doi.org/10.1111/j.1365-2672.2004.02398.x>.

Wong, C., Heuzenroeder, M.W., Flower, R. 1998. Inactivation of two haemolytic toxin genes in *Aeromonas hydrophila* attenuates virulence in a suckling mouse model. Microbiology. 144, 291–298. <https://doi.org/10.1099/00221287-144-2-291>.
