# Supplementary material for: Prevalence and genetic diversity of Aeromonas veronii isolated from aquaculture systems in the Poyang Lake area, China
Source: Front Microbiol. 2022 Dec 12;13:1042007. doi: 10.3389/fmicb.2022.1042007 (PMC9791064; doi:10.3389/fmicb.2022.1042007)
Supplement: Supplementary file 2 [file Table_2.DOCX]

Supplementary table 2 Primers and PCR conditions*^a^* for the antimicrobial resistance genes

| Target gene | Primers | Sequences (5’-3’) | Annealing  temperature (°C) | Amplicon  size (bp) | Reference |
| --- | --- | --- | --- | --- | --- |
| Extended-spectrum  β-lactamase | *BlaTEM* | F: CATTTCCGTGTCGCCCTTATTC  R: CGTTCATCCATAGTTGCCTGAC | 58 | 1,080 | Dahanayake et al., 2019 |
|  | *BlaSHV* | F: AGCCGCTTGAGCAAATTAAAC  R: ATCCCGCAGATAAATCACCAC | 58 | 795 |  |
|  | *BlaCTX-M* | F: CGCTTTGCGATGTGCAG  R: ACCGCGATATCGTTGGT | 52 | 550 |  |
|  | *BlaOXA* | F: GGCACCAGATTCAACTTTCAAG  R: GACCCCAAGTTTCCTGTAAGTG | 61 | 564 |  |
| Tetracycline resistance | *TetA* | F: GTAATTCTGAGCACTGTCGC  R: CTGCCTGGACAACATTGCTT | 62 | 1,000 |  |
|  | *TetB* | F: CTCAGTATTCCAAGCCTTTG  R: CTAAGCACTTGTCTCCTGTT | 58 | 400 |  |
|  | *TetE* | F: GTGATGATGGCACTGGTCAT  R: CTCTGCTGTACATCGCTCTT | 62 | 1,100 |  |
| Plasmid-mediated  quinolone resistance | *QnrA* | F: AGAGGATTTCTCACGCCAGG  R: TGCCAGGCACAGATCTTGAC | 56 | 580 |  |
|  | *QnrB* | F: GATCGTGAAAGCCAGAAAGG  R: ACGATGCCTGGTAGTTGTCC | 53 | 496 |  |
|  | *QnrS* | F: GCAAGTTCATTGAACAGGGT  R: TCTAAACCGTCGAGTTCGGCG | 56 | 428 |  |
| Aminoglycoside resistance | *strA-strB* | F: TATCTGCGATTGGACCCTCTG  R: CATTGCTCATCATTTGATCGGCT | 55 | 538 |  |
|  | *aphAI-IAB* | F: AAACGTCTTGCTCGA GGC  R: CAAACCGTTATTCATTCGTGA | 53 | 500 |  |
|  | *aac(3’)-IIa* | F: ATGGGCATC ATTCGCACA  R: TCTCGGCTTGAACGAATTGT | 55 | 749 |  |
|  | *aac(6’)-Ib* | F: TTGCGATGCTCTATGAGTGGCTA  R: CTCGAATGCCTGGCGTGTTT | 55 | 482 |  |
| integrons | *IntI* | F: ACGAGCGCAAGGTTTCGGT  R: GAAAGGTCTGGTCATACATG | 52 | 565 | Su et al., 2006 |
|  | *IntII* | F: GTGCAACGCATTTTGCAGG  R: CAACGGAGTCATGCAGATG | 52 | 403 |  |
| transposons | *TnpA* | F: TACTGCCGCGCATCAAGATC  R: AGAAAGTTCGTCCTGGGCTG | 59 | 411 | Dahlberg and Hermansson, 1995 |
| Transmissible plasmids | *Qu* | F: AGCGCCGTGCTGTCCGCBGCNTAYCG  R: CTCCGCAGCCTCGRCSGCRTTCCA | 64 | 179 | Alvarado et al., 2012 |
|  | *C12* | F:GCAGCACTGGAAAAATATCGCTATGGGGNATHAC  R: CAACGTGATAATCCCGTCRGGVCGRTG | 59 | 257 |  |
|  | *H11^b^* | F: CCGGCGTCGGAGAAYCAYCAYCA  R: AAGGTCGTATACCTTYCCKGCRTCRTG | 65 | 207 |  |
|  | *P12^c^* | F:GCACACTATGCAAAAGATGATACTGAYCCYGTTTT  R:AGCGATGTGGATGTGAAGGTTRTCNGTRTC | 53.8 | 189 |  |
|  | *F12* | F:AGCGACGGCAATTATTACACCGACAAGGAYAAYTAYTA  R: ACTTTTGGGCGCGGARAABTGSAGRTC | 55 | 234 |  |

*^a^*Unless specified otherwise, PCR thermocycle conditions for each reaction is as follows, initial denaturation at 94 ºC for 5 min, 30 amplification cycles consisting of 30 s at 95 ºC, annealing for 1 min, extension for 1 min at 72 ºC, and a final extension for 7 min at 72 ºC.

*^b^*Touchdown PCR: start at 65 ºC, ΔTa=−1 ºC per cycle, 15 cycles at 55 ºC.

*^c^*30 cycles, 1.5U Taq per reaction.

**References**

Dahanayake, P.S., Hossain, S., Wickramanayake, M.V.K.S., Heo, G.J., 2019. Antibiotic and heavy metal resistance genes in *Aeromonas* spp. isolated from marketed Manila clam (*Ruditapes philippinarum*) in Korea. J. Appl. Microbiol. 127, 941-952. <https://doi.org/10.1111/lam.13261>.

Alvarado, A., Garcillán-Barcia, M., Cruz, F., 2012. A degenerate primer MOB typing (DPMT) method to classify gamma-proteobacterial plasmids in clinical and environmental settings. PloS one 7, e40438. https://doi.org/10.1371/journal.pone.0040438.

Dahlberg, C., Hermansson, M., 1995. Abundance of Tn3, Tn21, and Tn501 transposase (*tnpA*) sequences in bacterial community DNA from marine environments. Appl. Environ. microbiol. 61, 3051-3056. https://doi.org/10.1128/aem.61.8.3051-3056.1995.

Su, J., Shi, L., Yang, L., Xiao, Z., Li, X., Yamasaki, S., 2006. Analysis of integrons in clinical isolates of Escherichia coli in China during the last six years. FEMS Microbiol. Lett. 254, 75-80. https://doi.org/10.1111/j.1574-6968.2005.00025.x.
