# Supplementary material for: Prevalence and genetic diversity of Aeromonas veronii isolated from aquaculture systems in the Poyang Lake area, China
Source: Front Microbiol. 2022 Dec 12;13:1042007. doi: 10.3389/fmicb.2022.1042007 (PMC9791064; doi:10.3389/fmicb.2022.1042007)
Supplement: Supplementary file 3 [file Table_3.DOCX]

Supplementary table 3 The antimicrobial susceptibility of 46 *Aeromonas veronii* strains to 16 antibiotics

|  | | *guidelines  (mm)  R I S | DC1S-1* | YXJ | PYG3 | DC2-2-1* | XG2S-2* | CCG1 | XYB | LJLC | LTFS5 | LTFS6 | LTFS7 | LTC2 | CCG | LTFS3 | LTFS4 | LTFS2 | BL16103 | XG2S-1* | LTJC | XYY | HML2 | PYLGC | JX1-2-2* | JX3-2-2* | XG1-2-1* | XG3-1-1* | YXCY | LTCC | FZ1SY* | .PYG2 | FZQP | DC2S-2* | FZ2N | FZ2SY* | JX3N-1* | FZ1SG* | YXC | JX1-2-1* | NX16104 | FZSY* | FZQPS* | LTFS4B | YH16103 | JX16102 | BL16104 | HMH2 |
| --- | --- | --- | --- | --- | --- | --- | --- | --- | --- | --- | --- | --- | --- | --- | --- | --- | --- | --- | --- | --- | --- | --- | --- | --- | --- | --- | --- | --- | --- | --- | --- | --- | --- | --- | --- | --- | --- | --- | --- | --- | --- | --- | --- | --- | --- | --- | --- | --- |
| Aminoglycosides | AK | ＜15~16＜ | 20 | 21 | 20 | 22 | 25 | 17 | 17 | 19 | 19 | 20 | 19 | 18 | 15 | 19 | 18 | 20 | 29 | 23 | 22 | 29 | 21 | 14 | 23 | 23 | 23 | 25 | 26 | 20 | 18 | 19 | 14 | 20 | 19 | 13 | 22 | 13 | 18 | 22 | 29 | 14 | 14 | 21 | 28 | 29 | 26 | 22 |
|  |  |  | S | S | S | S | S | S | S | S | S | S | S | S | I | S | S | S | S | S | S | S | S | R | S | S | S | S | S | S | S | S | R | S | S | R | S | R | S | S | S | R | R | S | S | S | S | S |
|  | N | ＜12~17＜ | 22 | 18 | 19 | 23 | 24 | 20 | 24 | 21 | 22 | 22 | 21 | 21 | 17 | 20 | 23 | 22 | 24 | 24 | 20 | 23 | 19 | 19 | 20 | 21 | 22 | 20 | 11 | 11 | 15 | 16 | 15 | 23 | 14 | 14 | 21 | 11 | 18 | 21 | 24 | 11 | 10 | 11 | 23 | 22 | 21 | 20 |
|  |  |  | S | S | S | S | S | S | S | S | S | S | S | S | S | S | S | S | S | S | S | S | S | S | S | S | S | S | R | R | I | I | I | S | I | I | S | R | S | S | S | R | R | R | S | S | S | S |
|  | GM | ＜13~14＜ | 18 | 16 | 16 | 24 | 25 | 17 | 22 | 20 | 19 | 20 | 21 | 21 | 13 | 19 | 20 | 22 | 27 | 23 | 22 | 25 | 19 | 18 | 26 | 25 | 25 | 26 | 19 | 19 | 12 | 18 | 20 | 22 | 13 | 20 | 23 | 21 | 23 | 26 | 28 | 21 | 22 | 20 | 27 | 22 | 25 | 19 |
|  |  |  | S | S | S | S | S | S | S | S | S | S | S | S | I | S | S | S | S | S | S | S | S | S | S | S | S | S | S | S | R | S | S | S | I | S | S | S | S | S | S | S | S | S | S | S | S | S |
|  | CTRX | ＜14~18＜ | 32 | 33 | 35 | 36 | 32 | 34 | 33 | 34 | 35 | 35 | 34 | 33 | 36 | 30 | 31 | 31 | 40 | 32 | 32 | 34 | 32 | 34 | 30 | 28 | 30 | 32 | 33 | 33 | 34 | 33 | 32 | 36 | 33 | 35 | 34 | 32 | 33 | 37 | 30 | 32 | 32 | 32 | 35 | 30 | 24 | 30 |
|  |  |  | S | S | S | S | S | S | S | S | S | S | S | S | S | S | S | S | S | S | S | S | S | S | S | S | S | S | S | S | S | S | S | S | S | S | S | S | S | S | S | S | S | S | S | S | S | S |
|  | AMP | ＜13~17＜ | 0 | 0 | 0 | 10 | 10 | 0 | 0 | 0 | 0 | 0 | 0 | 0 | 0 | 0 | 0 | 0 | 0 | 0 | 0 | 0 | 0 | 0 | 0 | 0 | 0 | 0 | 0 | 0 | 0 | 0 | 0 | 0 | 0 | 0 | 0 | 0 | 0 | 0 | 0 | 0 | 0 | 0 | 0 | 0 | 14 | 0 |
|  |  |  | R | R | R | R | R | R | R | R | R | R | R | R | R | R | R | R | R | R | R | R | R | R | R | R | R | R | R | R | R | R | R | R | R | R | R | R | R | R | R | R | R | R | R | R | I | R |
| Quinolones | NOR | ＜12~17＜ | 23 | 25 | 24 | 24 | 25 | 20 | 25 | 26 | 10 | 10 | 11 | 23 | 0 | 20 | 20 | 21 | 25 | 22 | 23 | 25 | 22 | 21 | 26 | 24 | 23 | 26 | 22 | 26 | 21 | 0 | 20 | 23 | 23 | 27 | 24 | 25 | 20 | 22 | 24 | 23 | 26 | 22 | 23 | 27 | 19 | 24 |
|  |  |  | S | S | S | S | S | S | S | S | R | R | R | S | R | S | S | S | S | S | S | S | S | S | S | S | S | S | S | I | S | R | S | S | S | S | S | S | I | S | S | S | S | S | S | S | S | S |
|  | OFX | ＜12~16＜ | 28 | 26 | 27 | 25 | 27 | 15 | 30 | 22 | 25 | 24 | 24 | 23 | 14 | 24 | 24 | 25 | 27 | 28 | 25 | 28 | 28 | 23 | 27 | 26 | 28 | 27 | 22 | 22 | 23 | 15 | 22 | 24 | 22 | 21 | 23 | 21 | 22 | 24 | 25 | 23 | 21 | 20 | 32 | 28 | 25 | 26 |
|  |  |  | S | S | S | S | S | I | S | S | S | S | S | S | I | S | S | S | S | S | S | S | S | S | S | S | S | S | S | S | S | I | S | S | S | S | S | S | S | S | S | S | S | S | S | S | S | S |
|  | ENR | ＜13~14＜ | 28 | 28 | 12 | 27 | 26 | 12 | 10 | 27 | 29 | 27 | 11 | 12 | 12 | 11 | 12 | 11 | 28 | 27 | 26 | 28 | 24 | 12 | 24 | 26 | 26 | 27 | 22 | 12 | 21 | 15 | 26 | 22 | 23 | 27 | 26 | 23 | 20 | 24 | 26 | 23 | 22 | 25 | 24 | 31 | 33 | 29 |
|  |  |  | S | S | R | S | S | R | R | R | S | S | R | R | R | R | R | R | S | S | S | S | S | R | S | S | S | S | S | R | S | S | S | S | S | S | S | S | S | S | S | S | S | S | S | S | S | S |
| Chloramphenicols | C | ＜12~18＜ | 20 | 24 | 11 | 22 | 23 | 26 | 28 | 30 | 29 | 29 | 28 | 27 | 26 | 29 | 29 | 28 | 45 | 33 | 25 | 29 | 30 | 12 | 29 | 30 | 33 | 35 | 21 | 25 | 26 | 26 | 25 | 28 | 19 | 19 | 26 | 22 | 24 | 26 | 33 | 18 | 20 | 27 | 35 | 35 | 15 | 22 |
|  |  |  | S | S | R | S | S | S | S | S | S | S | S | S | S | S | S | S | S | S | S | S | S | I | S | S | S | S | S | S | S | S | S | S | S | S | S | S | S | S | S | S | S | S | S | S | I | S |
|  | FFC | ＜12~18＜ | 22 | 28 | 0 | 29 | 30 | 26 | 31 | 33 | 31 | 29 | 30 | 27 | 28 | 28 | 28 | 11 | 29 | 33 | 32 | 30 | 31 | 10 | 28 | 27 | 28 | 30 | 24 | 28 | 29 | 25 | 27 | 30 | 25 | 24 | 29 | 28 | 28 | 27 | 33 | 25 | 26 | 29 | 35 | 39 | 22 | 26 |
|  |  |  | S | S | R | S | S | S | S | S | S | S | S | S | S | S | S | R | S | S | S | S | S | R | S | S | S | S | S | S | S | S | S | S | S | S | S | S | S | S | S | S | S | S | S | S | S | S |
| Macrolides | MID | ＜13~18＜ | 10 | 10 | 11 | 10 | 10 | 0 | 10 | 11 | 9 | 10 | 10 | 10 | 9 | 10 | 9 | 11 | 17 | 10 | 10 | 9 | 10 | 10 | 10 | 10 | 11 | 10 | 10 | 10 | 9 | 10 | 10 | 10 | 11 | 12 | 10 | 10 | 10 | 11 | 10 | 11 | 9 | 10 | 10 | 10 | 0 | 10 |
|  |  |  | R | R | R | R | R | R | R | R | R | R | R | R | R | R | R | R | I | R | R | R | R | R | R | R | R | R | R | R | R | R | R | R | R | R | R | R | R | R | R | R | R | R | R | R | R | R |
|  | ERY | ＜14~22＜ | 20 | 18 | 11 | 17 | 17 | 16 | 16 | 18 | 19 | 15 | 15 | 19 | 16 | 15 | 16 | 17 | 17 | 17 | 16 | 15 | 20 | 18 | 18 | 16 | 17 | 18 | 17 | 15 | 15 | 16 | 16 | 17 | 20 | 17 | 18 | 14 | 15 | 15 | 16 | 15 | 16 | 16 | 17 | 17 | 17 | 19 |
|  |  |  | I | I | R | I | I | I | I | I | I | I | I | I | I | I | I | I | I | I | I | I | I | R | I | I | I | I | I | I | I | R | I | I | I | I | I | I | I | I | I | I | I | I | I | I | I | I |
| Polymyxins | PB | ＜8~12＜ | 15 | 14 | 10 | 15 | 15 | 0 | 14 | 16 | 15 | 15 | 15 | 17 | 13 | 15 | 16 | 15 | 15 | 14 | 14 | 13 | 14 | 11 | 14 | 13 | 14 | 16 | 15 | 13 | 14 | 14 | 14 | 13 | 15 | 16 | 16 | 15 | 15 | 16 | 16 | 14 | 15 | 15 | 14 | 13 | 14 | 14 |
|  |  |  | S | S | I | S | S | R | S | S | S | S | S | S | S | S | S | S | S | S | S | S | S | I | S | S | S | S | S | S | S | S | S | S | S | S | S | S | S | S | S | S | S | S | S | S | S | S |
| Tetracyclines | TET | ＜14~19＜ | 23 | 12 | 0 | 10 | 27 | 12 | 28 | 28 | 29 | 22 | 27 | 24 | 10 | 30 | 30 | 31 | 29 | 27 | 28 | 26 | 32 | 10 | 33 | 32 | 28 | 25 | 29 | 26 | 26 | 27 | 28 | 22 | 12 | 9 | 30 | 31 | 25 | 26 | 22 | 14 | 21 | 24 | 12 | 32 | 0 | 24 |
|  |  |  | S | R | R | R | S | R | S | S | S | S | S | S | R | S | S | S | S | S | S | S | S | R | S | S | S | S | S | S | S | S | S | S | R | R | S | S | S | S | S | I | S | S | R | S | R | S |
|  | DOX | ＜13~15＜ | 28 | 22 | 9 | 23 | 28 | 12 | 27 | 26 | 26 | 27 | 26 | 28 | 12 | 29 | 23 | 24 | 25 | 26 | 26 | 28 | 26 | 12 | 24 | 24 | 25 | 26 | 28 | 29 | 9 | 12 | 14 | 25 | 14 | 26 | 27 | 28 | 29 | 28 | 29 | 28 | 28 | 27 | 29 | 28 | 29 | 28 |
|  |  |  | S | S | R | S | S | R | S | S | S | S | S | S | R | S | S | S | S | S | S | S | S | R | S | S | S | S | S | S | I | R | I | S | I | S | S | S | S | S | S | S | S | S | S | S | S | S |
| Sulfonamides | SXT | ＜10~16＜ | 29 | 28 | 0 | 29 | 27 | 0 | 26 | 30 | 30 | 31 | 29 | 29 | 28 | 28 | 29 | 26 | 27 | 25 | 28 | 29 | 30 | 0 | 34 | 31 | 31 | 26 | 27 | 30 | 28 | 29 | 28 | 28 | 26 | 25 | 28 | 28 | 27 | 29 | 30 | 25 | 24 | 25 | 29 | 30 | 30 | 28 |
|  |  |  | S | S | R | S | S | R | S | S | S | S | S | S | S | S | S | S | S | S | S | S | S | R | S | S | S | S | S | S | S | S | S | S | S | S | S | S | S | S | S | S | S | S | S | S | S | S |

Note: S, susceptible; I, intermediary; R, resistant. *Guidelines: drug resistance is less than the intermediate value, intermediate sensitivity is the intermediate value, and sensitivity is greater than the intermediate value; The number is the inhibition zone diameters in millimetres. AK , amikacin; N, neomycin; GM, gentamycin; CTRX; ceftriaxone; AMP, ampicillin; NOR, norfloxacin; OFX, ofloxacin; ENR, enrofloxacin; C, chloramphenicol; FFC, florfenicol; MID, midecamycin; ERY, erythromycin; PB, polymyxin-B; TET, tetracycline; DOX, doxycycline; SXT, trimethoprim/sulfamethoxazole.
